# A Lecture Introductory to the Course of Instruction in the Woman’s Hospital Medical College for the Session of 1870–71

**Published:** 1870-12

**Authors:** W. H. Byford

**Affiliations:** Professor of Clinical Surgery of Women


### CHICAGO MEDICAL EXAMINER.

N. S. DAVIS, M.D., Editor.

VOL. XI. DECEMBER, 1870. NO. 12

### Original Contributions.

### ARTICLE XXXVIII.

### A LECTURE INTRODUCTORY TO THE COURSE OF INSTRUCTION IN THE WOMAN'S HOSPITAL MEDICAL COLLEGE FOR THE SESSION OF 1870-71.

By W. H. BYFORD, A.M., M.D., Professor of Clinical Surgery of Women.

Ladies and Gentlemen:—The occasion of our assembling this evening is one of novelty, as well as interest. Our object is to inaugurate a Course of Lectures in a Woman's Medical College; to add another enterprise to the many in which Chicago is almost constantly embarking.

This is the first effort of the kind made in this city, and the interest attaching to it is commensurate with the importance of the welfare of the female sex. It is with a view to add to the usefulness, independence, and development of woman that this institution is designed and originated. The animus that actuates the founders of this institution is a hope to afford a new field for her capacities, a new object for her labor and ambition, an additional means by which she may elevate herself to a higher and more useful occupation, and, in every way, promote her welfare.

How far this hope is to be realized is to be determined by many unforeseen circumstances, but we believe it will be fulfilled, because of the merits of the cause in which we are engaged.

In many of the large cities in Europe and this country, provisions have been made for the professional education of women. In St. Petersburg, Vienna, Paris, London, and Edinburgh, in the old country,—in New York, Philadelphia, and Boston, in the University of Michigan at Ann Arbor, and probably elsewhere in this country,—in some of these places, the male and female students assemble in the same lecture-room.

This is the case, or has been, in Paris, to some extent, if not altogether.

In most places, however, the two sexes now are provided for separately.

In the great Austrian University, measures have been taken recently to have a course of instruction given to women separately, which is intended to be as complete as the general course has been heretofore. In Edinburgh, a similar arrangement has been entered into, and our young, but vigorous and enterprising neighbor, the Michigan University, has followed the example of her illustrious seniors in this respect. There are, also, separate colleges intended exclusively for educating women in the profession of medicine in London, St. Petersburg, New York, Boston, and Philadelphia; and the unfortunate French Empress had enlisted some of the eminent medical men of France in the project of forming a woman's medical college in Paris. She was overtaken by her misfortunes too soon, however, to accomplish her purpose, but it is hoped that the idea will be developed into fact by somebody in that great country, where there are growing reasons why women should have greater facilities for earning an honorable living.

It would seem, in reference to the professional education of women, the conviction was becoming general that they should have opportunities of pursuing their studies separately. Hence, in places where the experiment of assembling persons of the two sexes in the same apartments has been made, a change has been effected.

If indeed our manner of social education is not wrong in some of its most important particulars, we must recognize the indelicacy of teaching anatomy and physiology to men and women assembled in the same classes.

It is a subject of congratulation with the advocates of the education of women, that so many facilities have thus recently been added to those hitherto existing.

It is but a very short time since women were refused any opportunity of obtaining a good medical education. Now, however, there are medical schools enough to educate all who may wish to study medicine.

If we look at the history of the struggles of the three pioneer female students of medicine in this country, we can understand the terrible weight of prejudice that barred the road to their success. They were a brave little band, and deserve to be remembered as heroic examples in the road to progressive measures. Their names are: Elizabeth Blackwell, Sarah R. Adamson, and Emily Blackwell, all young girls with brave hearts. Miss Elizabeth Blackwell, who has grown gray under the weight of many cares and responsibilities, was the first woman who graduated in medicine in this country. After having studied for several years, she and her friends applied for her admission into many different medical colleges in the United States.

All the colleges in the large cities, as Boston, New York, and Philadelphia refused to accept female students. Some eminent professors in those institutions declared that it would be impossible for them to give an outspoken, full course of lectures to mixed classes. Others felt assured that it would be impossible to keep order at their lectures.

The three great objections—brought out by Miss Blackwell's efforts to be admitted to medical colleges—to women attending medical lectures with men were: 1st, that it was indelicate; 2d, would cause disorder; and, 3d, that women ought not to study medicine at all. These objections prevailed in all the institutions applied to, but the Geneva Medical College, then a flourishing school in Western New York. She attended two full courses of lectures in that institution, and graduated in a very creditable manner. And, by-the-way, this is the only regular medical college that has allowed a woman to attend two full courses of lectures in the same classes with men.

Although this was the termination of many of her difficulties, many prejudices awaited her in attempting to establish herself in the practice of medicine. She has succeeded, notwithstanding all the difficulties that beset her, and is now enjoying a very lucrative business, alongside some of the great men of the profession.

*Her* example has done more toward encouraging women to study the medical profession in this country than the influence of any other person.

When, a few years later, Miss Sarah R. Adamson was passing through the same ordeal, to obtain a degree in medicine, she found the doors of the Geneva Medical College closed against female medical students, and in all America there was not one regular medical college that would accept her as a candidate for its honors, and, contrary to her wishes and those of her friends, she was obliged to take the alternative of abandoning the hope of graduating, or enter the Central Medical College at Syracuse, New York, an eclectic institution. She graduated at that school in 1851, and, after having attended the Blockley Hospital for a considerable time, commenced practice, and is now enjoying a good income. She has honored herself, her sex, and her profession, by becoming a wife and mother, and is now Mrs. Dolley.

Miss Emily Blackwell is remembered by some of the citizens of Chicago as having attended her first course of lectures at Rush Medical College. This venerable school of the Northwest was the second regular medical college in the United States to admit women to its classes.

Miss Blackwell was not allowed, however, to attend her second course of lectures in Rush Medical College. The Illinois State Medical Society, constituted then as now, of an influential assemblage of medical men of the State, rebuked the College by passing a vote of censure for admitting female students to the privileges of the profession.

It was not until after many fruitless efforts to gain access to college teaching, that she was admitted to the Cleveland Medical College. She graduated in that institution, after having attended one course of lectures, in the Spring of 1854.

After these three heroines had intruded themselves into the profession—for they certainly entered it against the declared wishes of nearly the entire profession—they were in a better position to fight the battles necessary to destroy the prejudices of society against the professional equality of the sexes.

The result of their efforts strenuously and obtrusively continued to overcome the opposition of society, the timid submissiveness of their own sex, and the honest conviction of medical men of their own complete superiority, is that these women now live to see every facility for educating women for the profession that they could desire.

When the circumstances now inaugarated, have worked their fullest effects, then we will be able to judge of the fitness, capacity, and the propriety of women becoming doctors in medicine.

It now, in fact, as it never has been before, becomes their fault if women do not succeed in the practice of medicine, as they can at present, realize the advantages necessary to place them on an equal footing with men, in obtaining a medical education.

Woman's fitness to practise medicine has not been fairly tried. Hitherto, as far back as history carries us, her opportunites have been very inferior, and, as a consequence, she could not develope her capabilities in this respect.

At no time or place until now, has she enjoyed equality with man in the facilities for obtaining a medical education. Until now, if received into medical colleges, she was made aware in no very delicate manner, that her presence was an incubus upon the teaching, and she has been obliged to obtain her professional education under circumstances that necessarily rendered it deficient.

It is therefore unfair to judge of her possible achievements by the individuals thus educated, residing and practising in this or any other place. Not until she can have all the advantages of the other sex in private offices, in colleges, and hospitals, and receive the encouragement to develop her faculties fully, will it be reasonable and right to compare examples of the two sexes in the practice of medicine.

Heretofore she has been driven from one college to another when allowed to enter them at all; no medical college having the courage to execute its own wishes in receiving her, or entertaining her right to study and practise medicine.

None but heroines could surmount the difficulties constantly springing up in the way of her acquiring the knowledge necessary to practise medicine successfully.

Notwitstanding all these difficulties, we have some shining examples of success among female practitioners, in this country, as well as in the old world.

History too, is not entirely devoid of the evidence necessary to establish woman's claims to the right to be tried, before being denied the capacity to succeed in one profession. Some illustrious names stand recorded on her pages, that do honor to the profession. Two female authors in medicine have become classical by universal consent, they are Mesdames Boivin and La Chapellee. Although they may now be regarded as among the old authors, no work of any merit, is at present written upon Obstetrics or Diseases of Women and Children, but that contains quotations from both of them. The very latest and most popular work on Gynecology, contains quotations from both of them, as well as woodcuts representing diseased parts, methods of surgical procedure, and operations for the removal of morbid growths.

The members of the profession present, will understand the great honor such marks of approval from eminent men indicate.

Could any medical man be assured that his works and name would be held in the estimation which is attached to those of Madame Boivin, his ambition would be satisfied. Or that he would make as sure and durable an impression upon the literature of the profession, as Madame La Chapelle, he would hardly presume to hope for more.

It is not too much to assert that the impressions made upon the profession by these two immortal women are indellible, and that their names will never perish while the science of Gynecology is cultivated.

There have been many others, among the female sex, who have distinguished themselves, and there are others now laboring in our science, whose names will command enduring fame.

I could point to living women in this country who are prominent, and whose names will not be forgotten, while their eminent compeers continue to live in medical history.

This is surely an enviable record for woman, and will not fail to encourage the female medical students who are present to-night, to make such exertions as will secure like distinction and honor.

In regard to the fitness and capacity of women to compete with the opposite sex in the practice of the learned professions, we generally, I think, theorize ourselves into error. We assume that women are intellectually inferior to men, and thus reason upon the matter. The assumption being understood, our conclusions are certainly not ultimate.

If, *en masse*, there is any such difference, it can only be in the great aggregate.

If we take at random one hundred of each sex, and critically compare them, will anybody expect to find that there are not many women superior to many of the men in such an assemblage, and that with the same opportunities will they not excel accordingly. It is very evident, I think, in the sweeping decision of inferiority against the sex, we miss the opportunity to employ this talent and it is lost. Would it not be fair and right to throw sex out of consideration, and utilize the great amount of ability thus ignored?

Women thus superior, will acquire knowledge with more facility, and exercise it with more judgment than the great majority of men, and, it is reasonable to suppose, that impartial teaching and strict examinations will furnish sufficient test for this superiority.

If women cannot bear this test as well as men, if they cannot acquire a thorough knowledge of all the branches of medicine, they will thus be prevented from entering the profession; and I think no gallant leniency should be allowed to operate in their favor in the green room, and permit them to pass without they are as well prepared as male students.

The courage of women to meet the grave emergencies of the profession, is often called in question. But look for the proof. Is it in the facts of the case, or is it not entirely theoretical again? I do not think that it is even theoretically probable that women may not make bold surgeons.

Is that strength of nerve that guides the mind and steadies the hand of the surgeon in his great exhibition of skill in operations, born of the same animal courage that makes the male brute the master of his female companions? No. It is a professional courage. Take the knife from the surgeon of the army and place it in the hands of the commanding general, and witness the courage of the veteran as he proceeds to amputate a shattered limb and secure the artery through which the life-blood of his patient is being wasted. The military bravery that sustains him in the destruction of his kind, is not the sort of courage to serve him in the humane process of saving life.

The courage necessary in the sick-room, in the presence of appalling accidents and death, comes from our complete knowledge of the situation and familiarity with the circumstances, and when we learn and at once comprehend the state of affairs, we shall soon possess the courage to meet the emergency, and repair the damages, without quailing or fear enough to impair our usefulness on such occasions.

As an evidence that education and habit engender the courage of the surgeon, witness the first operation of the young man after he commences his professional career.

He is never cool and steady, and any sign of trepidation or unsteadiness he may betray at first should not be set down as proof that he cannot become a steady and reliable operator.

I know living surgeons, who stand high as daring and steady operators, who frequently perform the most difficult and hazardous operations with coolness and composure, who trembled, uncontrolably, at the cutting of a vein to bleed a man, or in lancing the gums of an infant, gave signs of trepidation in spite of their utmost efforts to conceal them. I am sure no woman could make a worse start in surgery than this.

I think there are some surgical operations to which the delicate tact of woman's hand is peculiarly adapted, and I will venture to say that in them she will some day achieve respectability at least.

It is not alone the female tyro who is afraid of blood. Only last winter, we had a gentleman in our college-class, who, although he repeatedly tried to familiarize himself with surgical operations, was as often driven from the room by a sense of faintness, as he witnessed the flow of blood under the surgeon's knife. And it is common to have one or more such young men as this in every medical class.

Women are the very best assistants in surgery. In New York and Boston, I know of two whose services are appreciated above those of any other assistants in the institution to which they are attached. I never knew a woman of intelligence and experience to shrink from the most trying positions near the surgeon, or fail him in his most difficult circumstances. It is stated that the first Cæsarean section ever performed in the Kingdom of Great Britain was done by Mary Donally, an Irishwoman, and I have been assured that the female practitioners and teachers in the medical colleges in the East operate with coolness and skill. I think, therefore, there is no good ground for doubting that women may be educated into the habits of bravery and steadiness of nerve necessary to meet all the emergencies of the profession, and that no greater number among them will fail in the hazardous duties of our calling than those of the opposite sex.

But their domestic duties are urged as an objection to educating women for physicians.

The present condition of society denies millions of them the opportunity of entering the domestic relations in a desirable manner.

This is a fact patent to the most superficial observer. The disparity of numbers in the two sexes is well-known, while increasing thousands of voluntary bachelors make the disparity operate more against the inequality of the sexes. I need not dwell upon the different modes in which the extravagance, frivolity, and unreasonable exactions of society make matrimony impossible to a large part of the men and women of great cities, especially; these are well understood. From these facts, many women are free to enter avocations that are incompatible with domestic duties. Others prefer a state of celibacy as affording a felicitous independence of the social oppression, of which they have a right to complain.

Such will often wed themselves to the professions as a matter of choice. The physical incapacity resulting from matrimonial relations does not obtain in their case.

It is objected, that it is a violation of propriety for women to practise medicine, and some of the cowardly opponents, in proof, sneer at the female physician behind pictures drawn by their own impure fancy. They imagine a delicate woman entering the chamber of a sick man, and administering to him under circumstances unheard of and unthought of, save by persons of such depraved character as themselves.

The impropriety does not consist really in the position woman will take, but in the false position assigned her by the objectors themselves. Respectable and modest women will not enter a promiscuous practice among all sorts of patients, but they will seek some appropriate speciality, and mostly confine themselves to the diseases of women and children. To this last specialty, they will generally confine themselves, and in it there is a demand for them.

How many ladies there are, who, under certain circumstances, would much prefer to be attended by their own sex, if they could feel assured of the skill and capacity of their attendants?

That there is a demand for practitioners of this sex is proven by the great number now employed, most of whom are uneducated and entirely incompetent for the position they assume.

Multitudes of women are now suffering from diseases that are rapidly making inroads upon their constitutions, because of the delicacy they feel in approaching medical men upon the subject of them. They often thus bear their ills for years, when they would willingly avail themselves of skilled attendance from their own sex.

I am aware, as it is often said, that many ladies choose to employ medical men to female physicians, but this preference arises from the confidence which the *hitherto* superior education of the former has given them and not from actual preference for the opposite sex in such relations.

For several years female medical students have been increasing in numbers. Encouraged by the success of those who have been successful and are enjoying the fruits of a good practice, they have been making application for admission to medical colleges for instruction; and, as is well known, several institutions have received them in their classes. After a trial of one term, the only regular medical college of this city, that has recently made the experiment, decided against the propriety and expediency of teaching all branches of our science to ladies and gentleman assembled in the same classes, and the officers were directed not to receive any more female matriculants.

The indefatigable, if not indomitable medical attendant of the Woman's Hospital of Chicago, finding her darling project for educating women for the profession thus unexpectedly frustrated, after due deliberation and consultation with her friends, inaugurated measures which have resulted in the enterprise we represent to-night.

This would have probably been impracticable, if the Woman's Hospital had not been in existence, and the profession has been witness to the efficiency of woman's labor in connection with it.

The Faculty of the College is composed largely of the consulting staff of that Hospital, and the members of it hope to be able to avail themselves of the advantages afforded by that instution for teaching.

It is deemed desirable, in fact, to identify the interests of the two, by making them mutually beneficial.

The plan of organization of the Woman's Hospital Medical College, is essentially that of the best medical institutions in the country. There are fourteen professorships, in which are included those of Anatomy, Physiology, Pathology, Chemistry, Materia Medica, Surgical Anatomy, Principles and Practice of Surgery, Theory and Practice of Medicine, Obstetrics, Diseases of Women and Children, Hygiene, Medical Jurisprudence, besides clinical teaching in all the practical branches.

The students will have access to the clinical teaching and lectures of the Cook County Hospital, and the Hospital for Women and Children. Two of the professors of this College are on duty at the County Hospital, Dr. Bogue in the Surgical Ward, and Dr. Fitch in the Obstetric and Gynecological Wards.

To such as are familiar with the subject, I think the plan of organization and clinical advantages afforded the students of this institution, will guarantee success and command approval.

The lecture term is twenty weeks long, and there will be six didactic lectures daily during that time, in addition to the clinical lectures given weekly at the County Hospital. It is also expected that the students will be able to attend the teaching of the Chicago Eye and Ear Infirmary, under the care of Prof. Holmes.

If I may be allowed to speak of the personnel of the Faculty, I think I may assure you that all the members of it are scientific and trustworthy. As will be seen, with one exception, they are men instead of women, for the reason that a sufficient number of the latter, with ample acquirements and experience, could not be procured to fill the places.

I feel assured that every one of them is in full sympathy with the movemement, and will labor industriously and earnestly to forward the interests and contribute to the instruction of the students who may entrust themselves to their guidance. Some of the professors are veterans, ripe in years and experience, and all are good, reliable teachers.

The requirements for graduation are such morally, and professionally, as are adopted by the medical colleges of the highest grade in the United States.

That they will be enforced, I have no reason to doubt, and I fully believe that all but thorough and competent students will fail to pass the examinations, and receive the honors of the institution. From these facts I think the community need have no fears, but that the graduates of this College will be competent to practise their profession efficiently, and in accordance with the most recent improvements in our science.

For this present session, we are provided with temporary rooms of the most economical character. They are, however, sufficient for the accommodation of the classes at present, but if prosperity attend our efforts, permanent apartments must be furnished in the future.

Shall we succeed?

It seems to me that this is an enterprise in which every true woman in Chicago should feel a deep and permanent interest. Can we not expect reasonably, that the ladies of this City will make the success of the Woman's Hospital Medical College, a common cause with us? We expect them to be stimulated by a determination to secure the success of this institution, for the purpose of demonstrating to skeptics, the capacity of woman to compete successfully for honor and emolument in the profession with men, that their own sex need but an opportunity to rival the best of the physicians of the present times.

We here, therefore, in the presence of this audience, formally dedicate the Woman's Hospital Medical College of Chicago, to the ladies of Chicago, and appeal to them to be our friends and foster-patrons. We ask them to take these sister institutions thus associated, under their guardianship, as affording one of the best means for advancing the interests of womankind in this locality.

In thus appealing to the ladies of Chicago, we do not intend to ignore the benevolence of the large-hearted gentlemen of the City, we hope they will remember with kindness this estimable cause, and come to the aid of their wives and daughters in this enterprise.

To the medical students present, who are visiting the City for the purpose of attending the Woman's Medical College; in behalf of Faculty, I am commissioned to tender a cordial welcome. I am authorized to assure you that all the members will interest themselves in your welfare during your stay here.

They desire to be considered your personal friends, who are ready to aid you in your studies, and advise you in all matters of interest to you.

It is believed that the arrangements made for your accommodation and teaching, will afford you every facility for obtaining a thorough knowledge of your profession. And it is hoped that each one of you will feel personally pledged, not merely to equal, but to excel in zeal and acquirements, the students assembled in other medical colleges in this City.

Your examples as students may stimulate others to higher attainments, correctness of deportment, and the manifestation of a laudable ambition in elevating themselves to high positions.

Let the responsibility you have taken upon you to represent your sex in stepping out from the ordinary walks hitherto allotted to you, be felt by each one of you, and so appreciated as to actuate you to earnest and energetic efforts in attaining to a thorough knowledge of the profession of medicine.

Thus you may honor your sex, your profession, and yourselves.

